# Supplementary material for: Low-stress livestock handling protects cattle in a five-predator habitat
Source: PeerJ. 2023 Feb 10;11:e14788. doi: 10.7717/peerj.14788 (PMC9924134; doi:10.7717/peerj.14788)
Supplement: Supplemental Information 3 [file peerj-11-14788-s003.docx]

Supplementary Materials for

Low-stress livestock handling protects cattle in a five-predator habitat

Naomi X Louchouarn, Adrian Treves

Correspondence to: [louchouarn@wisc.edu](mailto:louchouarn@wisc.edu)

**This PDF file includes:**

Table S1

R-code

**Table S1.** Expanded data table for use with R code listed below. Approaches are defined as visits per camera trap day. Indirect sign are calculated as visits per large carnivore survey. Seq refers to the treatment sequence of either pseud-control-treatment (PC-T) or treatment-pseudo-control (T-PC).

| **Herd** | **Seq** | **Treatment.Control** | **Phase** | **Wolf** | | **Bear** | | **Cougar** | | **Coyote** | |
| --- | --- | --- | --- | --- | --- | --- | --- | --- | --- | --- | --- |
|  |  |  |  | Approaches | Indirect sign | Approaches | Indirect sign | Approaches | Indirect sign | Approaches | Indirect sign |
| **1** | PC-T | PC | 1 | 0.0 | 0.20 | 0.022 | 0.80 | 0.0 | 0.0 | 0.0 | 0.40 |
| **1** | PC-T | T | 2 | 0.0 | 0.50 | 0.0 | 0.75 | 0.0 | 0.25 | 0.021 | 0.50 |
| **2** | PC-T | PC | 1 | 0.0 | 0.0 | 0.033 | 0.63 | 0.0 | 0.0 | 0.079 | 0.25 |
| **2** | PC-T | T | 2 | 0.0 | 0.50 | 0.0099 | 0.67 | 0.0 | 0.0 | 0.0 | 0.33 |
| **3** | PC-T | PC | 1 | 0.0 | 0.0 | 0.0 | 0.44 | 0.0 | 0.11 | 0.0091 | 0.44 |
| **3** | PC-T | T | 2 | 0.0 | 0.29 | 0.0076 | 0.71 | 0.0 | 0.0 | 0.023 | 0.43 |
| **4** | PC-T | PC | 1 | 0.0 | 0.0 | 0.016 | 0.29 | 0.0 | 0.0 | 0.0055 | 0.57 |
| **4** | PC-T | T | 2 | 0.0 | 0.0 | 0.0 | 0.80 | 0.0 | 0.0 | 0.024 | 0.60 |
| **5** | T-PC | T | 1 | 0.0 | 0.0 | 0.0066 | 0.14 | 0.0 | 0.0 | 0.0 | 0.14 |
| **5** | T-PC | PC | 2 | 0.0 | 0.29 | 0.0063 | 0.29 | 0.0 | 0.0 | 0.0 | 1.0 |
| **6** | T-PC | T | 1 | 0.0 | 0.25 | 0.014 | 1.125 | 0.0 | 0.0 | 0.0035 | 0.13 |
| **6** | T-PC | PC | 2 | 0.0 | 0.33 | 0.043 | 0.67 | 0.026 | 0.0 | 0.034 | 0.17 |
| **7** | T-PC | T | 1 | 0.0067 | 0.38 | 0.040 | 0.38 | 0.0 | 0.0 | 0.013 | 0.25 |
| **7** | T-PC | PC | 2 | 0.0 | 0.57 | 0.023 | 0.43 | 0.0 | 0.0 | 0.030 | 0.43 |
| **8** | T-PC | T | 1 | 0.0 | 0.25 | 0.0 | 0.25 | 0.0 | 0.0 | 0.067 | 0.25 |
| **8** | T-PC | PC | 2 | 0.0 | 0.67 | 0.0 | 0.67 | 0.0 | 0.0 | 0.0 | 0.0 |

**Table S2.** Table of range rider days (number of days with at least one range rider present) and range rider dose (summed number of range rider visits by phase. LC presence columns are based on number of days with at least one grizzly bear, black bear, wolf and coyote presence by phase is also included.

| **Herd** | **Range Rider Days** | | **Dose Effect** | | **Grizzly Bear** | | **Black Bear** | | **Wolf** | | **Coyote** | |
| --- | --- | --- | --- | --- | --- | --- | --- | --- | --- | --- | --- | --- |
|  | Phase 1 | Phase 2 | Phase 1 | Phase 2 | Phase 1 | Phase 2 | Phase 1 | Phase 2 | Phase 1 | Phase 2 | Phase 1 | Phase 2 |
| **1** | 4 | 22 | 4 | 33 | 4 | 0 | 3 | 1 | 1 | 2 | 2 | 3 |
| **3** | 7 | 20 | 15 | 32 | 6 | 2 | 3 | 1 | 0 | 4 | 13 | 2 |
| **3** | 4 | 12 | 4 | 15 | 0 | 1 | 5 | 1 | 0 | 2 | 5 | 5 |
| **4** | 6 | 13 | 6 | 21 | 1 | 0 | 0 | 0 | 0 | 0 | 5 | 7 |
| **5** | 10 | 5 | 12 | 5 | 1 | 2 | 3 | 2 | 0 | 2 | 2 | 6 |
| **6** | 16 | 10 | 19 | 12 | 4 | 6 | 3 | 1 | 1 | 2 | 2 | 2 |
| **7** | 16 | 12 | 24 | 11 | 5 | 3 | 4 | 1 | 6 | 5 | 8 | 8 |
| **9** | 14 | 6 | 18 | 4 | 0 | 0 | 1 | 0 | 1 | 2 | 5 | 0 |

**R-Code**

## 1. Standardizing the direct and indirect predator signs

subtract from the mean of all observations and divide by the SD of all observations. Do this for both response variables; approaches (trail camera data) and indirect sign (large carnivore surveys)

Data$Approaches<- Data$BA+Data$WA+Data$CA+Data$BBA
approaches_mean <- mean(Data$Approaches)
approaches_sd <- sd(Data$Approaches)
Data$Standard_Approaches <- (Data$Approaches-approaches_mean)/approaches_sd
Data$Indirect <- Data$BI+Data$WI+Data$CI
indirect_mean<- mean(Data$Indirect)
indirect_sd<-sd(Data$Indirect)
Data$Standard_Indirect<- (Data$Indirect-indirect_mean)/indirect_sd
hist(Data$Standard_Indirect)

Data$pooled <- Data$Standard_Approaches+Data$Standard_Indirect

## 2. Subset the data by treatment sequence for analysis

PCT<- subset(Data, Data$seq == “PCT”)

TPC<- subset(Data, Data$seq == “TPC”)

## 3. Examine each predator species for individual response to treatment

**Pooled LC**

Shapiro.test(Data$Pooled.Standardized)

#treatment effect

t.test(PCT$Pooled.treatmenteffect, TPC$Pooled.treatmenteffect, paired = T)

#period effect

t.test(PCT$Pooled.periodeffect, TPC$Pooled.periodeffect, paired = T)

**Bear**

Shapiro.test(Data$Bear.Standardized)

#treatment effect

t.test(PCT$Bear.treatmenteffect, TPC$Bear.treatmenteffect, paired = T)

#period effect

t.test(PCT$Bear.periodeffect, TPC$Bear.periodeffect, paired = T)

##

**Wolf**

shapiro.test(Data$Wolf.Standardized)

wilcox.test(PCT$Wolf.treatmenteffect, TPC$Wolf.treatmenteffect, paired = T)

wilcox.test(PCT$Wolf.periodeffect, TPC$Wolf.periodeffect, paired = T)

**Cougar**

shapiro.test(Data$Cougar.Standardized)

wilcox.test(PCT$Cougar.treatmenteffect, TPC$Cougar.treatmenteffect, paired = T)

wilcox.test(PCT$Cougar.periodeffect, TPC$Cougar.periodeffect, paired = T)

**Coyote**

Shapiro.test(Data$Coyote.Standardized)

#treatment effect

t.test(PCT$Coyote.treatmenteffect, TPC$Coyote.treatmenteffect, paired = T)

#period effect

t.test(PCT$Coyote.periodeffect, TPC$Coyote.periodeffect, paired = T)

## 4. Spearman’s Rank Correlations

#range rider data

RRDays <- Data$RRdays.Phase2-Data$RRdays.Phase1

RRDose <- Data$RRdose.Phase2-Data$RRdose.Phase1

#Grizzly Bear

GBChange <- Data$GB.Phase2-Data$GB.Phase1

cor.test(RRDays, GBChange, method="spearman")

cor.test(RRDose, GBChange, method="spearman")

#Black Bear

BBChange <- Data$BB.Phase2-Data$BB.Phase1

cor.test(RRDays, BBChange, method="spearman")

cor.test(RRDose, BBChange, method="spearman")

#Wolf

WChange <- Data$W.Phase2-Data$W.Phase1

cor.test(RRDays, WChange, method="spearman")

cor.test(RRDose, WChange, method="spearman")

#Coyote

CoyChange <- Data$Coy.Phase2-Data$Coy.Phase1

cor.test(RRDays, CoyChange, method="spearman")

cor.test(RRDose, CoyChange, method="spearman")
